# Traditional Medicine Extracts of *Gnidia sericocephala* and Product Nkabinde in HIV-1 Latency Reversal: Insights from J-Lat Subtype B and J-Lat Subtype C Models

**DOI:** 10.3390/ijms27031581

**Published:** 2026-02-05

**Authors:** Khanyisile Mngomezulu, Samukelisiwe Pretty Khathi, Siphathimandla Authority Nkabinde, Magugu Nkabinde, Mlungisi Ngcobo, Nceba Gqaleni

**Affiliations:** 1 Traditional Medicine, School of Nursing & Public Health, University of KwaZulu-Natal, Howard College, Durban 4001, South Africa; 210514678@stu.ukzn.ac.za (K.M.); nceba.gqaleni@ahri.org (N.G.); 2 Africa Health Research Institute (AHRI), Durban 4013, KwaZulu-Natal, South Africa; 3 HIV Pathogenesis Programme, University of KwaZulu-Natal, Durban 4041, South Africa; samkeezy@live.com; 4 Ungangezulu, Dundee 3000, South Africa; 5 Faculty of Health Sciences, Durban University of Technology, Durban 4000, South Africa

**Keywords:** HIV-1, African traditional medicine, latency, reactivation, latency reversal agents

## Abstract

The persistence of latent HIV-1 reservoirs in individuals on antiretroviral therapy (ART) remains a major barrier to cure, necessitating strategies such as “shock and kill” using latency-reversing agents (LRAs). However, current LRAs show limited clinical efficacy, highlighting the need for novel interventions. This study evaluated the in vitro latency-reversing potential of Product Nkabinde (PN) and *Gnidia sericocephala* using J-Lat A2 (subtype B) and J-Lat C clones T66 and T17 (subtype C) cells. Cell viability was assessed using flow cytometry with Live/Dead dye. Reactivation potential was further tested in combination with established LRAs: panobinostat, SAHA, and TNF-α. *G. sericocephala* induced dose-dependent latency reversal, with 26.1% of J-Lat A2 and 15.8% of J-Lat T66 cells GFP-positive at 106 µg/mL (*p* = 0.0001). Co-treatment with LRAs enhanced reactivation—34.6% with SAHA and 87.2% with TNF-α in J-Lat A2 cells, and 56.9% with SAHA and 65.4% with TNF-α in J-Lat T66 cells (*p* = 0.0001)—while maintaining cell viability above 90%. PN showed minimal activity (≤1.3% GFP-positive) and no effect in combination assays. Fractional inhibitory concentration index analysis revealed no synergistic interactions. Ex vivo, PN and *G. sericocephala* induced limited increases in HIV-1 gag RNA without substantial cytotoxicity. These findings demonstrate that *G. sericocephala* effectively reverses HIV-1 latency and potentiates TNF-α-induced reactivation, supporting its potential as a plant-derived LRA for future “shock and kill” HIV-1 cure strategies.

## 1. Introduction

The use of combination antiretroviral therapy (cART) has transformed the human immunodeficiency virus type 1 (HIV-1) infection into a manageable chronic disease. cART has decreased HIV-1/AIDS-related deaths as well as HIV-1 transmission [1,2]. As of the end of 2024, an estimated 31.6 million people living with HIV-1 globally (77%) were accessing cART treatment [3]. Although cART has been very effective in suppressing active virus replication to undetectable plasma viremic levels and promoting immune system reconstruction, it is not curative. Combination antiretroviral therapy (cART) must be continued indefinitely, as HIV-1 rapidly rebounds from persistent latent reservoirs upon treatment interruption and cannot be eliminated with currently available therapies [1,4,5,6,7]. Factors by which the virus persists in the presence of cART are uncertain and may involve multiple mechanisms, such as integration sites, epigenetic modifications, and transcriptional and post-transcriptional regulations [8,9].

A significant source of viral rebound is a reservoir of long-lived, resting memory CD4+ T lymphocyte cells that harbor integrated HIV-1 DNA within their genomes. These cells harbor transcriptionally silent HIV-1 proviruses that do not express viral antigens, thereby evading immune recognition and remaining unaffected by current antiretroviral therapies [10,11]. However, under circumstances that are not entirely clear but may include T-cell activation by cognate antigen, these latently infected cells can undergo transcriptional reactivation of integrated HIV-1 proviruses. Under effective antiretroviral therapy, this reactivation does not typically result in widespread de novo rounds of infection; instead, viral rebound is driven predominantly by transcriptional reactivation of long-lived, clonally expanded infected cells, while ongoing therapy prevents new infection events and reseeding of the reservoir [6,9,12,13]. The latent reservoir is the major barrier to HIV-1 eradication. Therapeutic approaches that will either reactivate the latent virus or induce permanent suppression of the latent reservoir are urgently needed for an HIV-1 cure [14,15]. One therapeutic approach used to identify and eliminate these latently infected CD4+ T cells is the “shock and kill” strategy. This approach involves reversing the latent viral state by employing latency-reversing agents (LRAs) that interfere with the cellular mechanisms believed to be associated with HIV-1 persistence. In principle, the reactivated latently infected cells should be cleared through viral cytopathic events or host immune system defenses. Concurrent maintenance of cART is essential to avoid new rounds of viral infection and re-seeding of the viral reservoir [16,17].

Latency-reversing agents are drugs that activate HIV-1 transcription, and a wide variety of the investigated compounds represent different functional classes [14,18]. The notable functional drug classes represent histone deacetylase (HDAC) inhibitors, protein kinase C (PKC) agonists, bromodomain extra-terminal motif inhibitors, cytokines, chemokines, and Toll-like receptor (TLR) agonists. These LRAs have been effective in activating HIV-1 provirus expression, as demonstrated in ex vivo and in vitro studies; however, only a few have advanced to clinical trials [19]. However, minimal changes in the latent HIV-1 pool were observed in clinical trials involving repeated sub-toxic dosing by these chemotherapeutic drugs [20,21,22]. Thus, major research efforts are directed toward developing clinically effective LRAs [19]. Several medicinal plants and their bioactive constituents have demonstrated potential as LRAs, often targeting key signaling pathways involved in HIV-1 transcriptional reactivation. Phorbol esters, such as phorbol 12-myristate 13-acetate (PMA), are potent PKC agonists that induce nuclear factor kappa B (NF-κB) and activator protein 1 (AP-1) signal transduction, leading to viral reactivation [23]. PMA has been widely used in vitro as a benchmark LRA due to its ability to induce T cell activation and HIV-1 transcription potently [24]. In addition, numerous cell models and methods have been developed to study HIV-1 latency and reactivation, including J-Lat cell lines expressing green fluorescence protein (GFP) [25]. However, these models do not display the same sensitivities to reactivation stimuli, with no single model accurately representing the behavior of latently infected patient cells [26].

Given the limited efficacy of broad-spectrum transcriptional activators, identifying potent and safe LRAs derived from medicinal plants is critical. More than 80 percent of the population in developing countries, such as Africa and Asia, use herbal medicines to manage their ailments, including HIV-1 and AIDS symptoms [27,28]. Several plants and their phytochemicals have been studied as potential LRAs for HIV-1 infection [29,30,31]. Phytochemicals in the diterpene class have been extensively investigated as LRAs. Prostratin, which is both a diterpene phorbol ester and a protein kinase C agonist, was first isolated from *Pimelea prostrata* (*Thymelaceae*) and demonstrates potent HIV-1 latency reversal properties [32,33,34]. Furthermore, other phytochemicals have been evaluated as potential HIV-1 latency reversal agents, including ingenol (diterpene) and procyanidin (flavonoid) [35,36,37]. Given this context, we examined crude extracts of a polyherbal traditional medicine formulation, Product Nkabinde. The formulation comprises four known medicinal plants described previously in [29,38,39] obtained from traditional health practitioners (THPs), Mr. S and Mr. M Nkabinde, of KwaZulu-Natal. PN is a traditional medicinal formulation prescribed by a registered traditional health practitioner, Mr. Sipathimandla Nkabinde, for the management of HIV-1-related illnesses. Unpublished observational clinical data collected during a period prior to the widespread availability of antiretroviral therapy in South African public health facilities included measurements of CD4+ T-cell counts and viral load in HIV-infected individuals receiving PN. These observations provided the rationale for subsequent scientific investigation of PN. Subsequent research studies by Tembeni et al. (2022), Setlhare et al. (2024), Mngomezulu et al. (2025), and Ugbaja et al. (2026) have further confirmed the potential of PN as a treatment for HIV-related ailments [29,38,39,40]. Clinical studies are currently pending ethical approval and are anticipated to commence in 2026. These four plants have been demonstrated to possess numerous biological activities, including antioxidant, antimicrobial, anti-inflammatory, antidiabetic, anticancer, anti-HIV-1, immune-modulating, wound-healing, and antispasmodic properties [29,41,42,43,44]. THPs have been using PN for the management of HIV-1 and AIDS-related symptoms for the last 30 years. The current study evaluated the effects of PN and *G. sericocephala* on HIV-1 latency reversal in J-Lat subtype B (clone A2) and J-Lat C (clones T66 and T17) cell models.

## 2. Results

### 2.1. PN and G. Sericocephala Display Low Toxicity in J-Lat Cells

**Figure 1:**
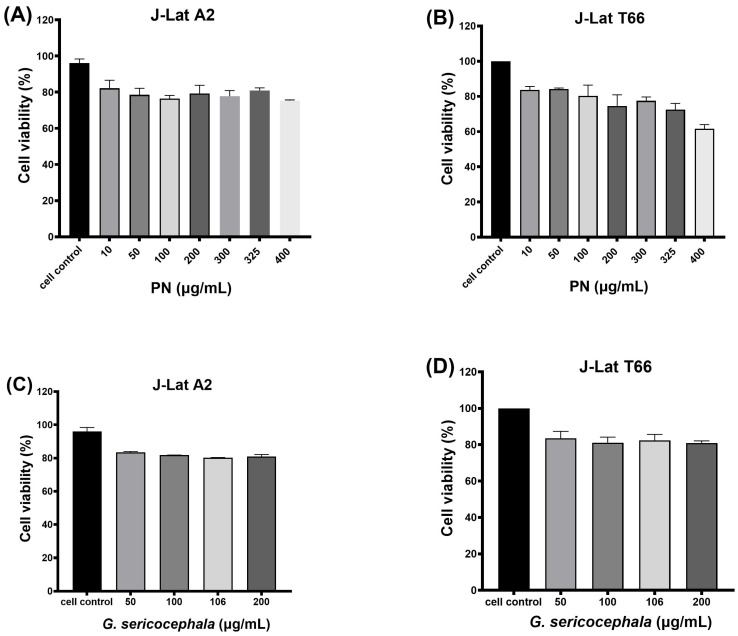
Cytotoxic effects of PN and *G. sericocephala* extracts on J-Lat cells. (**A**,**B**) Viability of J-Lat A2 and T66 cells following 24-h treatment with increasing concentrations (10–400 µg/mL) of PN. (**C**,**D**) Viability of J-Lat A2 and T66 cells treated with *G. sericocephala* extract (10–200 µg/mL) for 24 h. Cell viability was assessed using the Live/Dead assay. Data represent mean ± SEM of three independent experiments.

Cytotoxicity assays are critical for the preliminary safety assessment of plant-derived extracts, enabling the detection of potential toxic effects across various cell types. These evaluations inform the safe application of plant-based therapeutics by balancing efficacy with minimal toxicity [45,46,47]. Our data showed that the treatment of J-Lat A2 cells with PN at concentrations ranging from 10 µg/mL (82.2%), 50 µg/mL (78.6%), 100 µg/mL (76.5%), 200 µg/mL (79.3%), 300 µg/mL (77.8%), 300 µg/mL (81%), and 400 µg/mL (75.3%) resulted in minimal cellular cytotoxicity, with cell viability remaining above 75% across all concentrations (Figure 1A). In contrast, J-Lat T66 cells exhibited a dose-dependent decline in viability in response to PN at concentrations of 10 µg/mL (83.7%), 50 µg/mL (84.2%), 100 µg/mL (80.3%), 200 µg/mL (74.5%), 300 µg/mL (77.5%), 300 µg/mL (72.5%), and 400 µg/mL (61.7%), indicating moderate cytotoxicity in this cell type at higher concentrations (Figure 1B). Treatment with *G. sericocephala* extract at 50 µg/mL (83.3%), 100 µg/mL (81.8%), 106 µg/mL (80.2%), and 200 µg/mL (80.9%) showed a consistently low cytotoxic profile in both J-Lat A2 (Figure 1C) and C cells (Figure 1D), with cell viabilities maintained above 80% at all concentrations tested. Our data indicate that one of the plant extracts in PN is highly cytotoxic, as higher concentrations reduced cell viability to below 80%, whereas the *G. sericocephala* extract was less toxic, with cell viability remaining above 80% across all tested concentrations.

### 2.2. G. Sericocephala Extract Induces HIV-1 Expression in an In Vitro HIV-1 Latency Cell Culture Model

**Figure 2:**
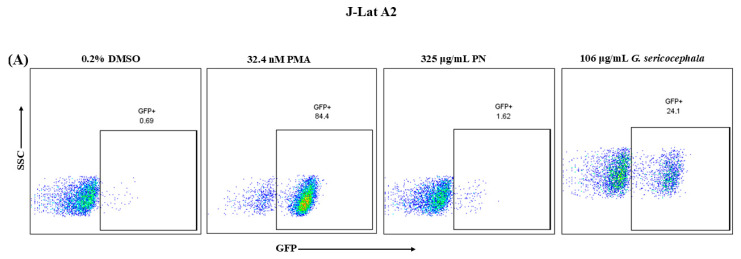

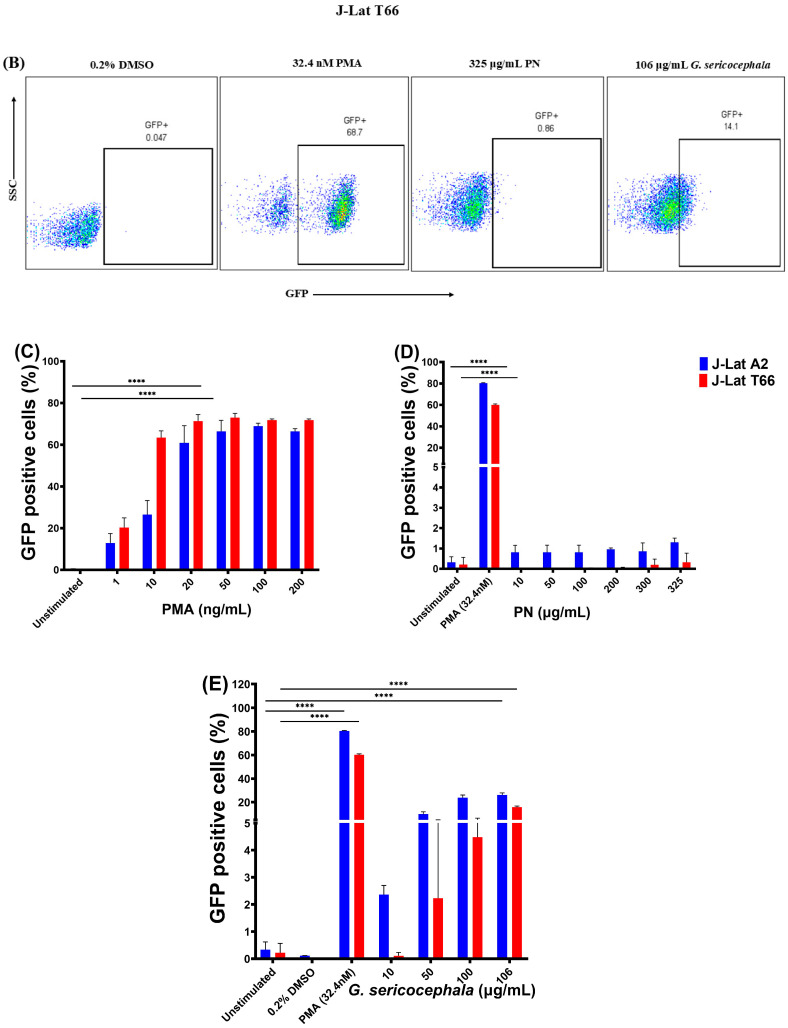
PN and *G. sericocephala* induce HIV-1 latency reversal in J-Lat A2 and T66 cells. Representative flow cytometry data showing latency reversal, as measured by GFP expression, in (**A**) J-Lat A2 and (**B**) J-Lat T66 cells. Values indicate the percentage of GFP-positive cells for each condition. (**C**) PMA showed a dose-dependent increase in GFP-positive cells in both J-Lat A2 and J-Lat T66. (**D**) PN showed low latent HIV−1 reactivation levels at all tested concentrations, with GFP expression remaining near background levels. (**E**) *G. sericocephala* induced a dose-dependent increase in GFP-positive cells, with the highest reactivation observed at 106 µg/mL in both J-Lat A2 and T66 cells. PMA (32.4 nM) robustly induced GFP expression and served as a positive control. Data are shown as mean ± SEM from three independent experiments. Statistical significance was assessed using two-way ANOVA with Tukey’s multiple comparisons test. **** *p* < 0.0001. The *y*-axis is segmented to allow visualization of both high PMA responses and lower-level treatment effects. Blue bars represent J-Lat A2 cells, while red bars represent J-Lat T66 cells.

Numerous latency-reversing agents (LRAs) have been evaluated for their ability to reactivate latent HIV−1, with some advancing to clinical trials; however, most have demonstrated limited efficacy in reducing the latent reservoir [21,48]. Previous studies have documented plants being used for HIV/AIDS management, and some as LRAs, including the *Euphorbiaceae* family [19,49]. We investigated the latency-reversing potential of PN and *G. sericocephala* using established J-Lat A2 and T66 cell models, which express GFP under the control of the HIV−1 LTR promoter upon reactivation. The flow cytometry analysis revealed distinct differences in latency reversal between the plant extracts and the positive control (Figure 2). Representative flow cytometry plots showed GFP-positive cells following treatment of J-Lat A2 cells with PMA, PN, and *G. sericocephala* (Figure 2A) and J-Lat T66 cells with the same agents (Figure 2B). The positive control, PMA, a well-known activator of the PKC pathway, demonstrated a dose-dependent decrease in the number of viable cells (1–200 ng/mL) (Supplementary Figure S1A,B). A dose-dependent increase in reactivated latent HIV−1 cells was observed in J-Lat A2 and J-Lat T66 cells, with 20 ng/mL showing 60.9% (*p* = 0.0001) of J-Lat A2 GFP-positive cells (95.8% viable cells) and 71.4% of J-Lat T66 cells (87.03% viable cells) (Figure 2C,D). PMA (32.4 nM) induced robust reactivation, resulting in GFP expression in 84.4% (*p* = 0.0001) of J-Lat A2 and 68.7% (*p* = 0.0001) of J-Lat T66 cells after 24 h of treatment (Figure 2D). PN exhibited very low levels of latency-reactivation potential, inducing only 1.62% GFP-positive J-Lat A2 cells and 0.86% in J-Lat T66 cells at the highest concentration tested (325 µg/mL) when compared to PMA (Figure 2D). Conversely, *G. sericocephala* extract demonstrated a significantly dose-dependent increase in GFP expression, with 106 µg/mL reactivating 24.1% (*p* = 0.0001) and 14.1% (*p* = 0.0001) of J-Lat A2 and T66 cells, respectively (Figure 2E). To further verify the observed low reactivation response in J-Lat T66 cells, an additional clone (J-Lat subtype C, clone T17) was included and tested under the same treatment conditions (Supplementary Figure S2A,B). Similar to the initial J-Lat T66 results, treatment with PN extract induced reduced GFP expression across all tested concentrations, confirming that the reduced latency reversal was not restricted to a specific J-Lat C clone. In contrast, *G. sericocephala* exhibited a dose-dependent increase in GFP-positive cells following treatment. Taken together, these findings suggest that *G. sericocephala* shows more potential in reversing HIV−1 latency than PN, although its activity remains lower than that of PMA. GFP-based latency reversal assays were performed independently of viability analyses, which were assessed in separate experiments.

### 2.3. Gnidia Sericocephala in Combination with Other LRAs Induces Reactivation of the HIV-1 Subtype C (T66) Virus

**Figure 3:**
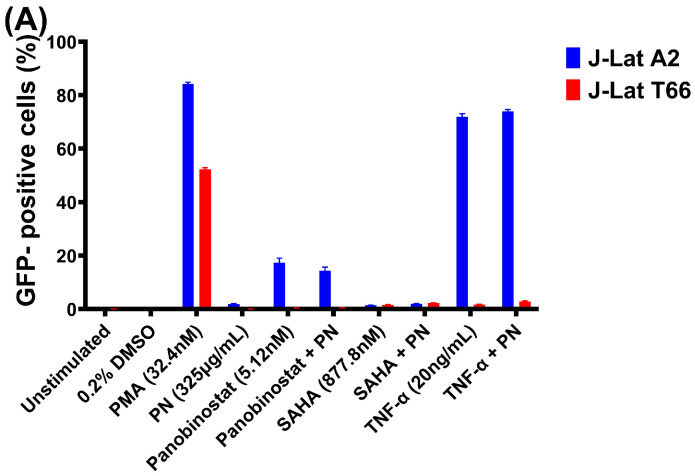

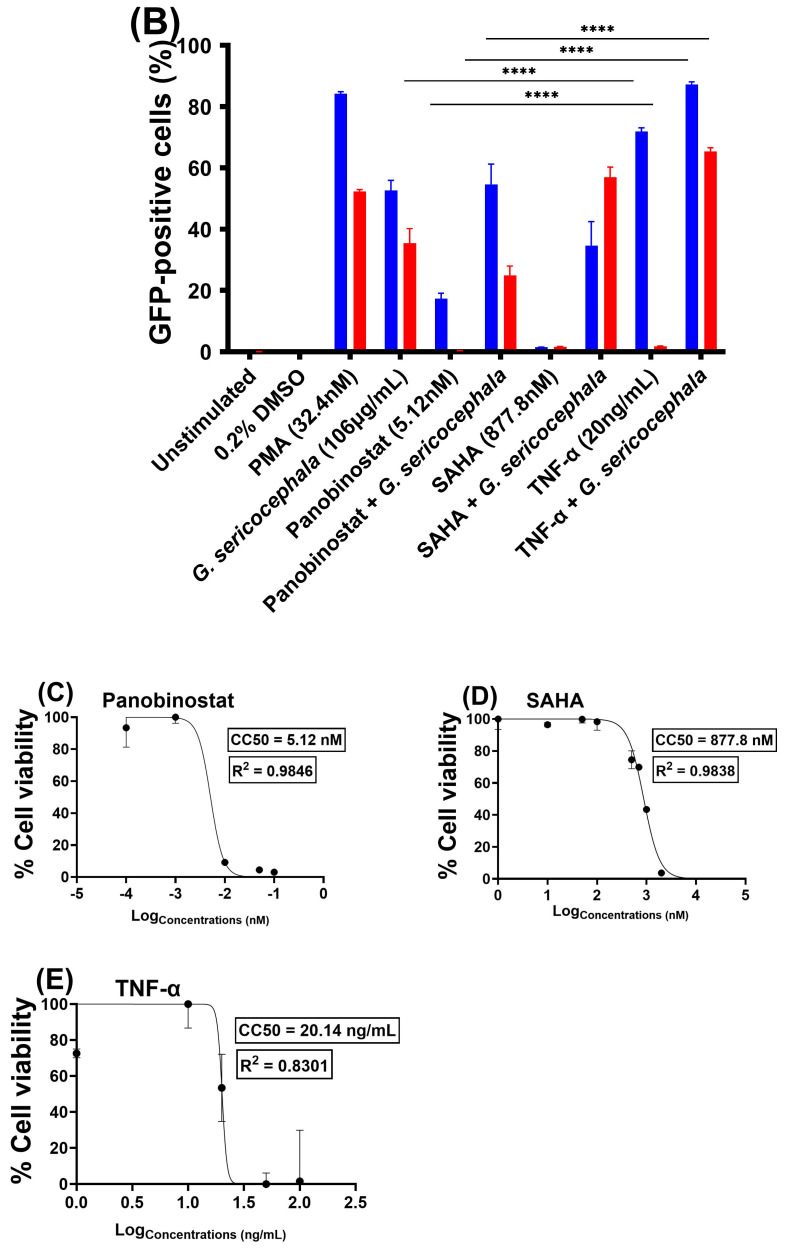

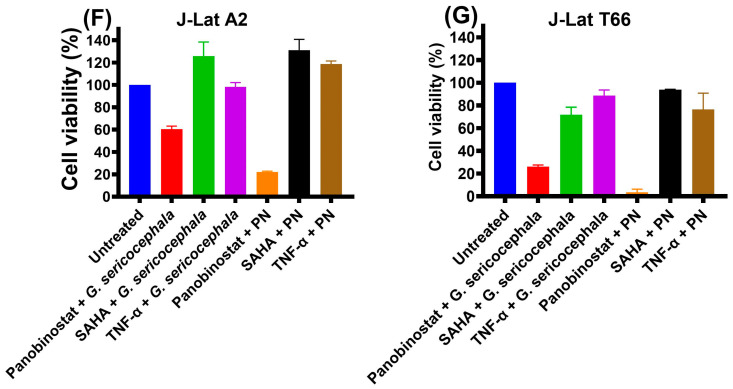
Combinatorial effects of PN and *Gnidia sericocephala* with latency-reversing agents (LRAs) on HIV−1 reactivation in J-Lat A2 and T66 cells. (**A**) PN in combination with panobinostat, SAHA, or TNF-α did not enhance HIV−1 reactivation compared to individual treatments, showing minimal GFP induction across both subtypes. (**B**) In contrast, *G. sericocephala* displayed strong synergistic effects with panobinostat and SAHA, significantly increasing the percentage of GFP-positive cells, particularly in J-Lat A2 cells. The combination with TNF-α also enhanced reactivation compared to single treatments. Data represent mean ± SEM of three independent experiments. Statistical significance was assessed using two-way ANOVA with Tukey’s multiple comparisons test. **** *p* < 0.0001. Bars are grouped by treatment conditions, with responses in the absence and presence of PN/*G. sericocephala* shown side-by-side to facilitate direct comparison. Blue bars represent J-Lat A2 cells, while red bars represent J-Lat T66 cells. Determination of cytotoxic concentrations of crude extracts on J-Lat cell lines. The graphical illustration of dose-dependent curves showing cell viability effects in cells treated with (**C**) Panobinostat, (**D**) SAHA, and (**E**) TNF-α. CC50 calculation was performed using the GraphPad Prism Version 10.6.1. (**F**,**G**) Cytotoxicity data for combination studies between medicinal plant extracts and LRAs. Cell viability was assessed using the CellTiter-Glo assay, which measures intracellular ATP levels. Raw luminescence values (relative light units, RLU) were first obtained for each condition. For comparative analysis, values were normalized to the untreated control (set to 100%), which can result in relative viability values > 100%.

HIV−1 latency is maintained by diverse mechanisms, and thus, single LRAs may be insufficient to achieve robust reactivation [14]. To explore potential synergistic effects, we assessed the activity of PN or *G. sericocephala* extracts in combination with other known LRAs: panobinostat (5.12 nM), SAHA (877.8 nM), and TNF-α (20 ng/mL), using J-Lat subtype B (A2) and subtype C (T66) models. PMA was used as a positive control in this experiment and was not combined with PN or *G. sericocephala*. In this study, PMA (32.4 nM) induced 84.2% GFP-positive cells in the J-Lat A2 cells and 52.3% in J-Lat T66 (Figure 3A,B). Initial treatments with individual LRAs showed that TNF-α induced the highest levels of GFP expression in J-Lat A2 (71.9%) compared to T66 (1.73%) cells, while panobinostat induced 17.3% (J-Lat A2) and 0.3% (J-Lat T66), and SAHA induced 1.5% (J-Lat A2) and 1.6% (J-Lat T66) GFP-positive cells (Figure 3A,B). Co-treatment with PN resulted in low reactivation for both J-Lat A2 (1.9%) and T66 (0.072%) (Figure 3A). In contrast, *G. sericocephala,* combined with panobinostat, SAHA, and TNF-α, significantly increased GFP expression in J-Lat T66 cells to 24.9% (*p* = 0.0001), 56.9% (*p* = 0.0001), and 65.4% (*p* = 0.0001), respectively. J-Lat A2 cells also showed significant increases with *G. sericocephala* combined with LRAs, particularly with TNF-α (87.2%, *p* = 0.0001) (Figure 3B). 

We evaluated the effects of these treatment combinations on cell viability in J-Lat A2 and J-Lat T66 cell lines, and the CC50 concentrations were determined for the tested LRAs (Figure 3C–E). Overall, most treatments, including *G. sericocephala*, PN, and their combinations with the LRAs, exhibited cell viability above 90% (Figure 3F,G). Raw luminescence values representing absolute viability are reported in Supplementary Table S1. However, treatment of J-Lat T66 with a combination of panobinostat and *G. sericocephala* markedly reduced viability by 33.8% (Figure 3G). Treatment of J-Lat T66 cells with PN and panobinostat reduced cell viability by 2.7% (Figure 3G). 

The FICI values for panobinostat and *G. sericocephala*, SAHA and *G. sericocephala,* and TNF-α and *G*. *sericocephala* were 4.42, 23.8, and 2.9, respectively, in J-Lat A2 cells, indicating no synergy. The FICI values for SAHA and *G. sericocephala* and TNF-α and *G. sericocephala* were 37.2 and 39.7, respectively, in J-Lat T66 cells, again indicating no synergy. The FICI values for all combinations assessed ranged from 1.6 to 84.01, indicating an indifferent effect (Supplementary Table S2). Taken together, these data suggest that the combination of PN and *G. sericocephala* extracts with LRAs shows significantly enhanced HIV−1 reactivation compared to individual LRAs; however, FICI analysis showed no evidence of synergistic interactions.

### 2.4. Gnidia Sericocephala and PN Induce Modest HIV-1 Transcription in Primary CD4+ T Cells from cART-Suppressed Individuals

**Figure 4:**
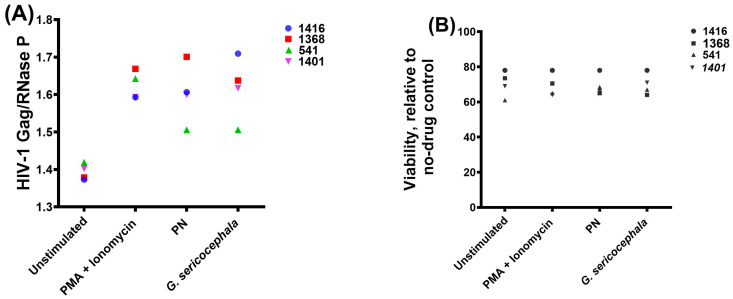
Ex vivo assessment of PN and *G. sericocephala* on HIV−1 transcription and cell viability in primary CD4+ T cells from cART-suppressed individuals. (**A**) Relative HIV−1 gag mRNA expression following treatment with PMA/ionomycin, PN, or *G. sericocephala*, quantified by seminested real-time PCR of four donors (1416, 1368, 541, and 1401). PMA/ionomycin induced the highest levels of gag expression, while PN and *G. sericocephala* resulted in modest, donor-dependent increases in gag mRNA levels compared to unstimulated controls. The Y-axis starts at 1.3 to allow individual data points to be clearly visible without overlapping. (**B**) Cell viability following 24 h treatment with PMA/ionomycin, PN, or *G. sericocephala* in CD4+ T cells. Viability is expressed relative to the unstimulated control. All treatments maintained cell viability above 61%, with most conditions exceeding 70%. Each symbol represents an individual donor.

Latency-reversing agents (LRAs) have been identified using in vitro models of HIV−1 latency and, in some cases, evaluated in clinical trials. However, assessing the extent of latency reversal achieved by candidate LRAs requires validation in ex vivo assays using cells from HIV−1-infected individuals. The development of such ex vivo assays is therefore critical to bridge findings from in vitro models to clinical application and to guide the design of future clinical trials [1]. To preliminarily assess whether PN and *G. sericocephala* could reverse HIV−1 latency ex vivo, resting CD4+ T cells were isolated from four individuals receiving suppressive cART with plasma viral loads below 50 copies/mL for at least three years (Table 1). Cells were treated for 24 h with PMA/ionomycin, PN, or *G. sericocephala* extract, followed by quantification of cell-associated HIV-1 gag RNA using a seminested real-time PCR assay. As expected, PMA/ionomycin induced gag mRNA expression in all donors, resulting in an average ~1.18-fold increase relative to unstimulated controls (Figure 4A). Treatment with PN and *G. sericocephala* resulted in modest increases in gag transcript levels in most donors, with average inductions of ~1.19-fold and ~1.21-fold, respectively. However, the magnitude of induction varied between individuals, and clear differences between treatment conditions and unstimulated controls were not consistently observed across all donors. *G. sericocephala* induced higher gag expression than PN in donors 1368 and 541, whereas PN showed greater activity in donor 1416, highlighting inter-individual variability in responsiveness. To evaluate potential cytotoxic effects, cell viability was assessed in parallel. All treatments maintained cell viability above 61%, with most conditions exceeding 70% viability (Figure 4B), indicating that the observed changes in gag expression were not associated with substantial cytotoxicity. Taken together, these ex vivo data indicate that PN and *G. sericocephala* induce modest and donor-variable HIV−1 transcription in resting primary CD4+ T cells from cART-suppressed individuals without substantial cytotoxicity.

## 3. Discussion

HIV−1 infection cannot be cured due to the persistence of latent HIV−1 reservoirs. The “shock and kill” strategy aims to reduce latent HIV−1 reservoirs through pharmacological reactivation of latent proviruses (“shock”), followed by clearance of reactivated cells primarily through immune-mediated mechanisms, while continued cART prevents new rounds of infection and limits reseeding of the reservoir (“kill”) [50,51]. Several latency-reversing agents have been investigated, with several PKC activators and HDAC inhibitors (e.g., SAHA, panobinostat, romidepsin, bryostatin, and disulfiram) undergoing clinical trials for the elimination of or reduction in latent reservoirs. Their efficacy in clinical settings has been limited due to incomplete latency reversal, potential toxicity, and variability in patient response [50,51]. Furthermore, given the multifactorial nature of HIV−1 latency, previous studies have reported that single LRAs are not effective at reversing latency ex vivo [50]. In addition, other studies have demonstrated that combination therapy comprising mechanistically distinct LRAs may be required to reverse latency robustly [51]. HIV−1 latency reversal in J-Lat cells using medicinal plants has shown promising results in vitro, highlighting the potential of natural compounds as latency-reversing agents (LRAs). Various studies have identified specific plant extracts and compounds that effectively reactivate latent HIV−1, providing a foundation for future therapeutic strategies.

In this study, we evaluated the latency-reversing potential of a crude preparation of PN and *G. sericocephala* extract (generated by ethanolic extraction and applied to cells in PBS containing 0.2% DMSO) in J-Lat A2 and J-Lat subtype C cell models, which serve as well-characterized in vitro systems for assessing HIV−1 latency and reactivation. This latent provirus also carries an integrated, but transcriptionally latent, HIV−1 provirus with a green fluorescent protein (GFP) reporter instead of *nef* accessory gene [52,53]. As a result, HIV−1 latency reversal was monitored by the percentage of GFP-positive cells using flow cytometry, consistent with published data [50,54]. The control LRA PMA, a phorbol ester that reverses HIV−1 latency through activation of the protein kinase C (PKC) pathway, was included as a positive control due to its potent and reproducible latency-reversing activity across multiple in vitro latency models. PMA also serves as a benchmark for defining the maximal level of HIV-1 reactivation achievable in a given system; combining it with other latency-reversing agents (LRAs) would compromise its role as a reference [26,55]. Our results show that PMA exhibited significant expression of GFP-positive cells at the known effective concentration. For the traditional medicine extracts, PN exhibited low reactivation activity for both J-Lat A2 and J-Lat subtype C clones (T66 and T17) when compared to PMA. Interestingly, using the J-Lat A2 and J-Lat subtype C clones (T66 and T17) cell model of latency, we established that *G*. *sericocephala* reactivates HIV−1 in a dose-dependent manner.

Our findings demonstrate that *G*. *sericocephala* effectively reverses HIV−1 latency in both J-Lat cell lines, with a dose-dependent increase in GFP-positive cells, indicating viral reactivation. Notably, *G. sericocephala* exhibited superior latency reversal activity compared to PN, which showed minimal reactivation across all tested concentrations, suggesting that its mechanism of action may not significantly impact latency-associated pathways in J-Lat cells. The observed reactivation potential suggests that *G. sericocephala* contains bioactive compounds capable of modulating key transcriptional pathways, possibly through PKC activation, NF-κB induction, or chromatin remodeling mechanisms. A previous study showed that compounds including yuanhuacine A and yuanhuacine isomeric mixture from *G. sericocephala* were able to induce significant HIV−1 latency reversal in J-Lat 10.6 cells, achieving 65.5% and 58.2% GFP expression, respectively, at 0.15 µM, compared to prostratin [29]. In another study, Nkwelle et al. [2] investigated the latency reversal properties of six extracts obtained from *Croton oligandrus* and the crude extract, Mukungulu, isolated from *Croton megalobotrys,* all found in Africa. Their results showed that the most potent extract from *C. oligandrus*, UB/CA12, induced 69.7 ± 7.1% GFP expression at 1 μg/mL, whilst Mukungulu extract demonstrated a dose-dependent expression of GFP in J-Lat cells with 76.3 ± 8.2% (10 µg/mL), compared to prostratin, which showed a dose-dependent expression of GFP across multiple concentrations with 75.2 ± 1.8% at 11.6 µg/mL. Furthermore, although our study did not determine the mechanism by which *G. sericocephala* induces latent HIV−1 in vitro, Tembeni et al. [3] demonstrated that compounds, including yuanhuacine A and a yuanhuacine isomeric mixture from *G. sericocephala*, function as PCK activators. To be clinically applicable, effective LRAs should show minimal cytotoxicity [19]. Our viability assays revealed that *G. sericocephala* exhibited moderate cytotoxicity. However, cell viability remained relatively high at concentrations where latency reversal was most significant (10–106 µg/mL), indicating a favorable therapeutic window. Furthermore, the extract was prepared by 50% ethanol extraction followed by spray-drying, resulting in negligible residual ethanol in the final product.

The establishment and maintenance of HIV−1 latency involve multiple mechanisms; therefore, a single LRA may not be sufficient to reverse latency in all cells. Almost all the identified candidate LRAs were only minimally active in resting CD4+ T cells from infected individuals [19,50]. However, when mechanistically distinct LRAs were tested together, multiple combinations were identified that effectively reversed latency [51,56]. Quantifying the combined effect of two or more LRAs requires an understanding of the impact of each drug alone [51]. We investigated the combinatorial effects of *G. sericocephala* and PN with other mechanistically distinct LRAs, including the two HDAC inhibitors, panobinostat and SAHA, which induce histone acetylation, leading to a more relaxed chromatin structure that allows for the transcription of the HIV−1 genome, thus reactivating latent HIV−1 from infected cells. Additionally, TNF-α, a pro-inflammatory cytokine that activates NF-κB signaling independently of PKC, was included in the combination treatment studies [57,58,59]. Although formal synergy was not confirmed by FICI analysis, the data demonstrated a clear combinatorial effect, particularly in J-Lat subtype C (T66) cells. Combinations involving TNF-α, panobinostat, and SAHA resulted in significantly higher frequencies of GFP-positive cells compared to individual latency-reversing agents alone. This trend suggests a potential enhancement of latency reversal when these agents are used together, warranting further mechanistic investigation to determine whether cooperative or context-dependent interactions underlie the observed increases in HIV−1 transcriptional activation. Notably, for panobinostat and SAHA, the observed increases in GFP expression following co-treatment with *G. sericocephala* appear to be primarily driven by the plant extract itself, as their individual effects were minimal and the combination did not result in a substantial enhancement. This suggests that *G. sericocephala* alone may be responsible for most of the latency reversal activity observed in these conditions.

The most notable increase in latency reversal was observed with the combination of *G. sericocephala* and TNF-α, resulting in a 65.4% reactivation rate in the J-Lat T66 and T17 clones (both containing subtype C LTRs) and an 87.2% response in the J-Lat A2 clone (subtype B). These results suggest that *G. sericocephala* may act through a complementary mechanism that enhances the efficacy of established LRAs, although the effects observed are specific to the tested clones and cannot be directly generalized to all subtype C or B viruses. Although the FICI analysis did not support a synergistic interaction, the pronounced reactivation trend is compelling and warrants further investigation. One possible explanation is that *G. sericocephala* may modulate cellular signaling pathways that converge on NF-κB activation, a central driver of HIV−1 transcriptional reactivation. NF-κB signaling is a well-established pathway activated downstream of PKC agonists, such as phorbol 12-myristate 13-acetate (PMA), as well as pro-inflammatory cytokines like TNF-α. In resting CD4+ T cells, NF-κB exists as an inactive p65/p50 heterodimer sequestered in the cytoplasm through binding to IκBα. Upon stimulation, PKC agonists induce IKK-mediated phosphorylation of IκBα, leading to its ubiquitination and proteasomal degradation. This process liberates the p65/p50 heterodimer, allowing its nuclear translocation and subsequent displacement of inhibitory p50/p50 homodimers at the HIV−1 long terminal repeat (LTR), ultimately promoting transcriptional activation [8,57,60].

The moderate effect seen with HDAC inhibitors (panobinostat and SAHA) further supports the notion that *G. sericocephala* influences chromatin remodeling or transcription factor recruitment to complement epigenetic modifiers. Our results are in line with previous studies that have explored plant-derived compounds as latency-reversing agents. For instance, Bullen et al. [4] and Darcis et al. [5] demonstrated that various HDAC inhibitors and PKC agonists effectively reverse HIV−1 latency, particularly when used in combination. Additionally, studies by Jiang et al. [6] and Laird et al. [7] investigated and identified multiple plant-based LRAs with potential latency reversal effects, and these studies support the role of natural compounds in HIV−1 eradication strategies. Furthermore, a study by Wang et al. [8] highlighted that combinatorial approaches using different mechanistic LRAs lead to enhanced HIV−1 reactivation, reinforcing the combinational effects observed in this study. These studies can validate our findings collectively and suggest that *G. sericocephala* could be a novel addition to the growing list of plant-derived LRAs with promising latent reversal activity. Furthermore, combinations of *G. sericocephala* and PN with various LRAs led to significantly enhanced levels of HIV−1 reactivation compared to treatment with LRAs alone. However, combining plant extracts with established LRAs, such as panobinostat, may allow the use of lower effective doses, thereby minimizing the cytotoxic side effects observed. Nonetheless, FICI analysis indicated no evidence of either synergistic or additive interactions. These results suggest that while the combinations may improve reactivation outcomes, the effects likely stem from the engagement of parallel or cooperative pathways rather than direct pharmacological synergy.

HIV−1 subtype C and subtype B differ in the number of NF-κB binding sites in their long terminal repeat (LTR) promoter regions. A typical HIV−1 subtype C LTR contains three or four canonical NF-κB binding sites [61,62], whereas most major subtypes, including subtype B, contain two genetically identical NF-κB sites that constitute the viral enhancer [63,64,65]. The additional NF-κB sites in subtype C are thought to contribute to stronger transcriptional activity, enhancing its replicative fitness, and may partly explain its global predominance in India, Southern Africa, Ethiopia, and Southern Brazil [61,62,66,67]. Interestingly, in our study, the J-Lat A2 clone (subtype B) was more readily reactivated than the J-Lat subtype C clones (T66 and T17), despite having fewer NF-κB binding sites. This suggests that clone-specific factors, such as proviral integration site and chromatin environment, can influence latency reversal beyond the number of NF-κB motifs in the LTR. *G. sericocephala* reversed latency in both the J-Lat A2 (type B) and T66 (type C) clones; however, as each J-Lat clone carries a single, unique proviral integration, these observations may reflect clone-specific features rather than general subtype-specific effects. Further studies with multiple type C isolates are required to confirm broader activity.

Interestingly, while *G. sericocephala* is a component of PN, the extract alone exhibited measurable latency-reversing activity, whereas PN showed low levels of reactivation activity. This suggests that other compounds of PN may antagonize or dilute the latency-reversing effect of *G. sericocephala*. Moreover, although *G. sericocephala* has been reported to inhibit HIV−1 replication [29,38], these effects are not necessarily contradictory. Latency reversal reflects activation of transcription from the integrated provirus, whereas inhibition of viral replication may involve interference with viral protein function or virion production [68]. Therefore, it is possible that *G. sericocephala* can promote proviral transcription in HIV−1 latent cells while restricting productive viral replication.

While our current study demonstrates latency reversal in J-Lat cell lines, the effects of PN and *G. sericocephala* in primary T cells remain to be fully established. Recent studies have shown that crude extracts from medicinal plants can reverse HIV−1 latency ex vivo in cells from individuals receiving combination antiretroviral therapy (cART) [29,69,70]. In this study, we conducted preliminary ex vivo experiments to evaluate the ability of PN and *G. sericocephala* to induce HIV−1 transcription in resting CD4+ T cells isolated from individuals receiving suppressive cART. While both extracts demonstrated latency-reversing activity in J-Lat cell line models, their effects in primary cells were modest and variable and did not differ consistently from unstimulated controls. Such findings are consistent with previous reports demonstrating that latency reversal observed in transformed cell lines often fails to translate robustly to resting primary CD4+ T cells ex vivo [71]. Primary T cells harbor latent HIV−1 within a transcriptionally restrictive chromatin environment that limits proviral accessibility and gene expression, resulting in low baseline transcriptional activity and making modest increases in viral RNA challenging to detect in ex vivo assays, particularly in small donor cohorts. Additionally, assays using crude plant extracts at single concentrations may further limit the magnitude of detectable responses [50,72,73,74]. Importantly, PN and *G. sericocephala* treatment did not result in substantial cytotoxicity, suggesting that the modest increases in gag mRNA were not driven by nonspecific cell activation or loss of viability. This supports the notion that these extracts may contain biologically active components that modulate HIV−1 transcription without inducing overt toxicity [75]. Although the ex vivo results do not demonstrate robust latency reversal, they provide valuable insight into the limitations of translating in vitro findings to primary cell systems and underscore the need for further optimization.

While this study offers valuable insights, it is very important to acknowledge certain limitations. Although cell viability was assessed, additional mechanistic studies are necessary to characterize the cytotoxic effects of *G. sericocephala* and PN, particularly through quantification of apoptosis and necrosis to support their safety profiles for potential preclinical development. For the synergy assays, the use of multiple concentrations to assess the effect of these combinations at lower concentrations can shed further insights into their potential applications for in vivo models. In addition, while the FICI method provides an initial understanding of potential combinatory effects, more detailed analyses, including the Bliss Independence model, can be used to characterize these LRA interactions better. In addition, the study assessed latency reversal at a single time point of 24 h post-treatment, which does not provide information on the duration or sustainability of PN and *G. sericocephala* effects. Assessing latency reversal at different time points would be beneficial in understanding the kinetics of latency reversal and the potential long-term consequences of treatment to cells. The current study also did not analyze the integration sites to understand why HIV−1 J-Lat subtype B (A2) cells were more reactivatable than J-Lat subtype C (T66 and T17) cells. Lastly, in this study, latency reversal was evaluated using the J-Lat cell line model of latency, which provides a well-established and reproducible platform for mechanistic investigations and initial screening. Although these experimental models do not fully capture the complexity of HIV−1 latency in primary human CD4+ T cells, they provide proof-of-concept evidence supporting the latency-reversing activity of *G. sericocephala* and the more limited activity of PN in established in vitro models. Preliminary ex vivo assessment in resting primary CD4+ T cells revealed modest and variable effects, underscoring the challenges of translating latency-reversal activity from cell line systems to primary cells and highlighting the need for optimized assay conditions. Future studies focusing on refined ex vivo models and in vivo validation will be critical to determine the translational potential of these extracts and to clarify their role in HIV−1 latency reversal strategies.

## 4. Methods and Materials

### 4.1. Cells, Drugs, and Reagents

The J-Lat subtype B (clone A2) [76] and J-Lat subtype C (clones T66 and T17) [77] lines used in this study were obtained from Dr. Paradise Madlala (Human Pathogenesis Program). J-Lat A2 cells contain a replication-incompetent HIV−1 construct in which the viral LTR drives expression of GFP and Tat in the absence of additional viral proteins [76]. In J-Lat A2, transcriptional inference drives latency due to integration into the highly expressed *UTX* gene [78]. The HIV−1 subtype C lentiviral vector, C731CC, was created by modifying an HIV−1 subtype B vector [76] to replace the original subtype B LTRs and *tat* gene with their subtype C counterparts, ensuring that both the HIV−1C Tat protein and GFP are under the control of the HIV−1C 5′ LTR (subtype C LTR-Tat-IRES-GFP) [77]. These cells were cultured in R10 medium (Gibco, Thermo Fisher Scientific, Waltham, MA, USA) supplemented with 10% fetal bovine serum (FBS) (Gibco BRL, Life Technologies), 5% Penicillin Streptomycin (Pen-Strep) (Sigma-Aldrich, St. Louis, MO, USA), 1% HEPES (Gibco, Thermo Fisher Scientific, Waltham, MA, USA), and 1% L-Glutamine (L-Glut) (Sigma-Aldrich, St. Louis, MO, USA) and were grown at 37 °C and 5% CO_2_. The U.S. Food and Drug Administration (FDA)-approved drugs used as LRAs—panobinostat (HDAC inhibitor), phorbol 12-myristate 13-acetate (PMA; PKC activator), SAHA/vorinostat (HDAC inhibitor), and TNF-α (NF-κB activator)—were obtained commercially from Sigma-Aldrich (St. Louis, MO, USA). Panobinostat and SAHA/vorinostat were supplied as powders and dissolved in dimethyl sulfoxide (DMSO) to generate concentrated stock solutions. For cell-based assays, all DMSO-solubilized compounds, including *G. sericocephala* and LRAs, were diluted in PBS such that the final DMSO concentration in culture did not exceed 0.2%. A matched 0.2% DMSO vehicle control was therefore included for all treatments containing DMSO.

Peripheral blood mononuclear cells (PBMCs) were obtained from four people living with HIV−1 (PLWH) who had been maintained on combination antiretroviral therapy (cART) for at least four years. These participants were enrolled in the Females Rising through Education, Support, and Health (FRESH) cohort, an ongoing prospective study established in Durban, KwaZulu-Natal, South Africa, in 2012 [79]. The FRESH cohort recruits young women aged 18–23 years who are initially HIV−1 negative but at high risk of infection to characterize early events in HIV−1 acquisition. Participants attend twice-weekly sessions that combine socioeconomic empowerment classes with routine finger-prick blood collection for HIV−1 testing. To date, more than 2500 women have been enrolled, and more than 95 cases of acute HIV-1 infection have been identified. Written informed consent was obtained from all participants, and the study was approved by the University of KwaZulu-Natal Biomedical Research Ethics Committee, as referenced in a study by our group [80]. CD4+ T cells were isolated from these PBMCs and rested in R10 medium.

### 4.2. Plant Material and Preparation of Plant Extract

The traditional medicine product (PN) investigated in this study corresponds to a Type C extract, defined as a polyherbal formulation derived from lesser-studied botanical species that are not listed in pharmacopeias or widely commercialized. PN comprises four medicinal plants: *Sclerocarya birrea* subsp. *afra* (*Anacardiaceae*), *Gnidia sericocephala* (*Meisn.*) *gilg ex Engl.* (*Thymelaeaceae*), *Senna italica* subsp. *italica* (*Fabaceae*), and *Pentanisia prunelloides* subsp. *prunelloides* (*Rubiaceae*). Detailed descriptions of PN and *G. sericocephala* and their composition, ethnobotanical sourcing, phytochemical analysis, and preparation have been reported previously [29,38,39].

For experimental use, PN was filter-sterilized and freeze-dried using a benchtop freeze dryer (VirTis, SP Scientific, Warminster, PA, USA) to obtain a powdered extract, which was stored at −20 °C until use. For experiments, PN was reconstituted to a final concentration of 10 mg/mL in phosphate-buffered saline (PBS; Thermo Fisher Scientific, Carlsbad, CA, USA) and filter-sterilized. PN was fully soluble in PBS due to its preparation by boiled-water extraction. *Gnidia sericocephala* extract was prepared by Afriplex Pharmaceuticals (https://afriplex.co.za) in Paarl, South Africa, via spray-drying of a 50% water–ethanol extract. For cell-based assays, *G. sericocephala* extract was initially solubilized in DMSO due to limited solubility in PBS and subsequently diluted with PBS to achieve a working concentration of 10 mg/mL, resulting in a final DMSO concentration of 0.2% in cell culture. DMSO is commonly used to dissolve poorly water-soluble compounds, acting as a solvent (vehicle) to deliver them to cells. The same 0.2% DMSO control was therefore used for all *G. sericocephala* treatment conditions to monitor any biological activity of this vehicle control.

### 4.3. In Vitro Cytotoxicity Assay

The cytotoxic effects of PN and *G. sericocephala* on J-Lat A2 and J-Lat T66 cells were evaluated after 24 h of treatment. Cell viability was assessed using the Live/Dead™ Fixable Blue Dead Cell Stain Kit (Thermo Fisher Scientific, Carlsbad, CA, USA) and measured on a BD FACSymphony™ A5 flow cytometer (Becton Dickinson, San Jose, CA, USA). Data were analyzed using FlowJo v10.8.1 (BD Biosciences, Ashland, OR, USA). Briefly, 2.5 × 10^5^ J-Lat A2 and J-Lat T66 cells were seeded into 96-well microtiter plates in a total of 250 µL R10 medium and treated with varying concentrations of PN (10–325 µg/mL) and *G. sericocephala* (50–200 µg/mL). Untreated controls (J-Lat A2 and J-Lat T66 cells in medium only) were included. Plates were incubated for 24 h at 37 °C with 5% CO_2_. Following incubation, cells were centrifuged at 3500× *g* for 5 min, and the supernatant was discarded. The cell pellets were resuspended, and 300 µL of Live/Dead stain (1:1000 dilution) was added to each well. The mixture was incubated at 4 °C for 30 min. After staining, the cells were washed with 900 µL of PBS and centrifuged at 3500× *g* for 4 min. Supernatant was removed, and the cell pellet was fixed in 250 µL of BD Cytofix™ fixation buffer (BD Biosciences, San Jose, CA, USA) for 20 min at 4 °C. Cells were then washed with 900 µL PBS, centrifuged at 3500× *g* for 4 min, and resuspended in 350 µL of FACS buffer. Samples were transferred to FACS tubes for flow cytometric analysis. Live/dead staining distinguishes viable from non-viable cells based on membrane integrity. Live cells were identified by exclusion of DAPI-positive events following initial gating on FSC-A/SSC-A to exclude debris and subsequent doublet discrimination. This assay was used exclusively to quantify cell viability and was not intended to assess HIV-1 reactivation. The flow cytometric gating strategy is shown in Supplementary Figure S3A,B.

### 4.4. In Vitro Latency Reversal Assay

The ability of PN, *G. sericocephala* extracts, and PMA to reverse HIV−1 latency was assessed using J-Lat A2 and J-Lat subtype C clones (T66 and T17) cell lines. Cells were seeded into 96-well plates at a density of 2 × 10^5^ cells per well in 180 µL of R10. Treatments were prepared separately at the indicated concentrations of PN (10–325 µg/mL), *G. sericocephala* (10–106 µg/mL), and PMA (1–200 ng/mL). Twenty-microliter (20 μL) aliquots of each treatment were added to the corresponding wells, resulting in a final culture volume of 200 µL per well. Cells were incubated with treatments for 24 h at 37 °C in 5% CO_2_. All treatments were added from concentrated stock solutions such that final concentrations were identical across all experimental conditions. Since *G. sericocephala* was dissolved in 0.2% DMSO, this concentration of DMSO was included as a control, and the untreated control was included. Following incubation, cells were harvested by centrifugation at 1500× *g* for 10 min and fixed with 2% paraformaldehyde (PFA). HIV−1 latency reversal was quantified by measuring GFP fluorescence, which is under the control of the HIV−1 LTR, using a BD LSR II flow cytometer (Becton Dickinson, San Jose, CA, USA). Data were analyzed using FlowJo v10.8.1 (BD Biosciences, San Jose, CA, USA). Live, single cells were identified based on forward and side scatter (FSC/SSC) properties and doublet exclusion before GFP analysis. All experimental conditions were performed in triplicate.

### 4.5. Combination Latency Reversal and Viability Assays

J-Lat A2, J-Lat T66, and J-Lat T17 cells were seeded at a density of 2 × 10^5^ cells per well in 180 µL of R10 medium in 96-well flat-bottom plates. Treatments were prepared from concentrated stock solutions and added immediately after seeding in a fixed volume of 20 µL per well, resulting in a final culture volume of 200 µL under all experimental conditions. Latency-reversing agents (LRAs) were applied either individually or in combination. For combination treatments, agents were mixed at a 1:1 volume ratio before being added to the cells. Stock concentrations were adjusted to ensure that each compound reached its intended final concentration without altering the total treatment volume or concentration. Final concentrations tested included PN (206.9 µg/mL), *G. sericocephala* (47.05 µg/mL), panobinostat (0.7 nM), SAHA/vorinostat (3.1 nM), and TNF-α (4.3 ng/mL). Untreated and the 0.2% DMSO control wells were included. Cells were incubated for 24 h at 37 °C in a humidified atmosphere containing 5% CO_2_.

Following incubation, cells were harvested and fixed with 2% paraformaldehyde for flow cytometric analysis. Latency reversal was quantified as the percentage of GFP-positive cells using a BD LSR II flow cytometer (Becton Dickinson, San Jose, CA, USA) and analyzed with FlowJo v10.8.1. Cell viability during the extract/LRA combination treatments was assessed in parallel using the CellTiter-Glo^®^ Luminescent Cell Viability Assay (Promega, Madison, WI, USA), which quantifies ATP content as an indicator of metabolic activity. Since LRAs can be highly cytotoxic, the effects of combining LRAs with the extracts were unknown; therefore, the CellTiter-Glo assay was performed to confirm that the selected treatment concentrations were non-toxic. Absolute viability values were calculated as the percentage of viable cells relative to the total cell count, and normalized values were expressed relative to untreated controls (set at 100%).

The fractional inhibitory concentration index (FICI) was used to determine if the drug combination effect is synergistic, additive, or antagonistic using the following formula:

$$\mathrm{FICI}=\frac{\mathrm{MICAB}}{\mathrm{MICA}}+\frac{\mathrm{MICBA}}{\mathrm{MICB}}$$

where MICAB has the minimum inhibitory concentration (MIC) of drug A tested in combination, MICA is the MIC of drug A tested alone, MICBA is the MIC of drug B tested in combination, and MICB is the MIC of drug B tested alone. Values of FICI ≤ 0.5, FICI > 0.5 but ≤ 4.0, and FICI > 4.0 determine synergistic, additive, and antagonistic interactions, respectively [81].

### 4.6. Measurement of HIV-1 Latency Reversal Ex Vivo

PN and *G. sericocephala* were evaluated for their ability to reverse HIV−1 latency using primary human CD4+ T cells. Cryopreserved peripheral blood mononuclear cells (PBMCs) were obtained from four people living with HIV−1 (PLWH) receiving suppressive combination antiretroviral therapy (cART), defined as plasma viral loads below 50 copies/mL for at least three years. CD4+ T cells were isolated from PBMCs using the EasySep™ Human CD4+ T Cell Enrichment Kit (STEMCELL Technologies, Vancouver, BC, Canada), as previously described by our group [77]. Purified CD4+ T cells were resuspended at a density of 4.6 × 10^6^ cells/mL and rested in R10 medium at 37 °C in a humidified incubator with 5% CO_2_ for 4 h. Cells were then treated for 24 h with PMA/ionomycin (100 ng/mL and 0.1 µg/mL, respectively), PN (206.9 µg/mL), or *G. sericocephala* extract (47.05 µg/mL). Following incubation, viable cells were counted using trypan blue exclusion, pelleted by centrifugation at 1700 rpm for 10 min, and transferred into 1.5 mL microcentrifuge tubes for downstream RNA extraction.

#### Seminested Real-Time PCR Assay to Quantify Cell-Associated RNA

Quantification of HIV−1 gag transcripts was performed using a seminested real-time PCR assay as previously described [82,83]. Total RNA was extracted from 3.42 × 10^6^ pelleted CD4+ T cells using the RNeasy Plus Mini Kit (Qiagen, Germantown, MD, USA), according to the manufacturer’s instructions. RNA concentration and purity were assessed spectrophotometrically, and only samples with OD_260_/OD_280_ ratios ≥ 1.90 were included in downstream analyses.

To eliminate residual HIV−1 DNA, 25 µL of eluted RNA from each sample was treated with DNase I (Invitrogen, Thermo Fisher Scientific, MA, USA) before reverse transcription. Complementary DNA (cDNA) synthesis was performed using the iScript™ cDNA Synthesis Kit (Bio-Rad Laboratories, CA, USA). Briefly, 1 µg of total RNA was reverse-transcribed in a final reaction volume of 20 µL containing 4 µL of 5× iScript reaction mix, 1 µL of iScript reverse transcriptase, RNA template, and nuclease-free water. Reverse transcription was carried out under the following conditions: 25 °C for 5 min, 46 °C for 20 min, and enzyme inactivation at 95 °C for 1 min.

HIV−1 gag transcripts were amplified using a two-round PCR approach. The first-round PCR was performed in a 25 µL reaction volume containing 5 µL of cDNA template, 20 mM Tris-HCl (pH 8.3), 50 mM KCl, 2 mM MgCl_2_, 0.4 mM dNTPs, 1 U AmpliTaq DNA polymerase (Applied Biosystems, Foster City, CA, USA), and 50 ng of each primer: GAG1 (Forward: 5′-TCAGCCCAGAAGTATACCCATGT-3′) and SK431 (Reverse: 5′-TGCTATGTCAGTTCCCCTTGGTTCTCT-3′). Cycling conditions were 94 °C for 3 min, followed by 15 cycles of 94 °C for 30 s, 55 °C for 30 s, and 72 °C for 1 min.

Products from the first PCR were used as templates for the second, seminested real-time PCR. Quantitative PCR was performed using primers targeting the HIV-1 gag region (gag-forward: 5′-CAAGCAGCCATGCAAATGTT-3′; gag-reverse: 5′-ATGTCACTTCCCCTTGGTTCTC-3′; and Fam-labeled HIV−1 gag probe:/56-FAM/5′-CCTGGTGCAATAGGCCCTGC-3′/3BHQ_1/) according to the manufacturer’s instructions (Thermo Fisher Scientific, Carlsbad, CA, USA). Amplification was carried out under the following conditions: initial activation at 50 °C for 2 min and 95 °C for 20 s, followed by 40–50 cycles of 95 °C for 30 s and 60 °C for 60 s using the LightCycler 480 (Roche Diagnostics, Basel, Switzerland). All samples were run in duplicate. HIV−1 gag RNA levels were normalized to the cellular RNase P reference gene (Thermo Fisher Scientific, Carlsbad, CA, USA). Experimental design, assay performance, and data analysis were conducted in accordance with MIQE guidelines.

### 4.7. Statistical Analysis

All data were analyzed using GraphPad Prism version 10.6.1 (GraphPad Software Inc., San Diego, CA, USA). All experiments were performed in biological triplicate, and data are presented as mean ± standard error of the mean (SEM). Cytotoxicity curves and CC50 values for the latency-reversing agents (panobinostat, SAHA, and TNF-α) were generated using non-linear regression analysis with a sigmoidal dose-response model. Differences in HIV−1 latency reversal, quantified as the percentage of GFP-positive J-Lat A2 and T66 cells, were analyzed using two-way analysis of variance (ANOVA) with treatment and cell line as independent factors, followed by Tukey’s multiple comparisons test. A significance threshold of *p* < 0.05 was applied for all statistical tests, with additional significance levels reported at *p* < 0.01, *p* < 0.001, and *p* < 0.0001 where indicated.

## 5. Conclusions

The ethanolic extract of *Gnidia sericocephala* effectively reactivated latent HIV−1 in J-Lat models, with a more prominent response in the subtype B (J-Lat A2) cells than in the J-Lat subtype C clones (T66 and T17). Inclusion of an additional J-Lat subtype C clone (T17) confirmed that the limited reactivation observed in subtype C was consistent across clones. The extract exhibited minimal cytotoxicity, maintaining cell viability above 80% at all tested concentrations, suggesting good biocompatibility. Importantly, *G. sericocephala* enhanced HIV−1 reactivation when combined with established latency-reversing agents. These results suggest that G. sericocephala contains bioactive constituents that can modulate HIV−1 latency, with activity influenced by the viral subtype and treatment context.

These findings indicate that *G. sericocephala* contains bioactive constituents capable of reversing HIV−1 latency, although its efficacy appears to vary between viral subtypes. The study provides preliminary evidence supporting the potential of this traditional medicinal plant as a source of novel latency-reversing agents. Further investigation using primary CD4+ T cells and mechanistic analyses is warranted to validate and characterize its molecular mode of action.

In summary, this study highlights *G. sericocephala* as a promising latency-reversing agent, demonstrating robust HIV−1 reactivation in both subtype B and C latency models, particularly in combination with established LRAs. These findings warrant further investigation in ex vivo and in vivo models to assess therapeutic efficacy, safety, and inter-individual variability to advance *G. sericocephala* toward clinical application in HIV−1 cure strategies.

## Figures and Tables

**Table 1:** Clinical characteristics of patients in this study.

Patient ID	Gender	Age (Years)	CD4 Count (Cells/μL)	Antiretroviral Therapy	Viral Loads (cps/mL)
127-33-1865-1416	Female	26	1227	FDC pill + RAL	<20
127-33-1809-1368	Female	21	1485	FDC pill + RAL	<20
127-33-0745-541	Female	26	1128	FDC pill	<20
127-33-1858-1401	Female	26	1478	FDC pill + RAL	<20

RAL: Raltegravir; FDC: fixed-dose combination.

## Data Availability

The original contributions presented in this study are included in the article/Supplementary Materials. Further inquiries can be directed to the corresponding author.

## Supplementary Materials

The following supporting information can be downloaded at: https://www.mdpi.com/article/10.3390/ijms27031581/s1.
