# Supplementary material for: Traditional Medicine Extracts of Gnidia sericocephala and Product Nkabinde in HIV-1 Latency Reversal: Insights from J-Lat Subtype B and J-Lat Subtype C Models
Source: Int J Mol Sci. 2026 Feb 5;27(3):1581. doi: 10.3390/ijms27031581 (PMC12898761; doi:10.3390/ijms27031581)
Supplement: Supplementary file 1 [file ijms-27-01581-s001.zip › ijms-4089668-supplementary.pptx]

## Slide 1
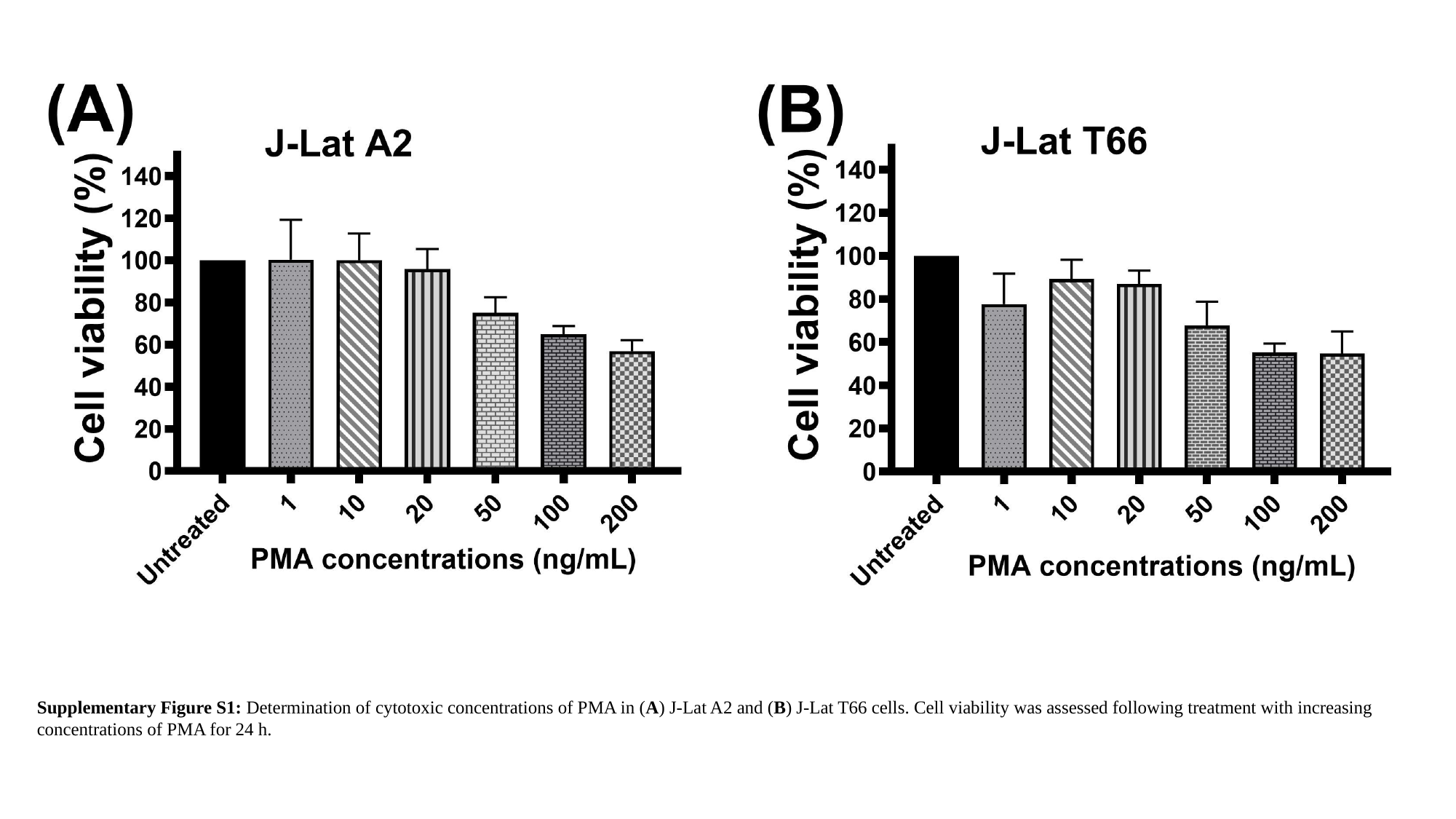

Supplementary Figure S1: Determination of cytotoxic concentrations of PMA in (A) J-Lat A2 and (B) J-Lat T66 cells. Cell viability was assessed following treatment with increasing concentrations of PMA for 24 h.

## Slide 2
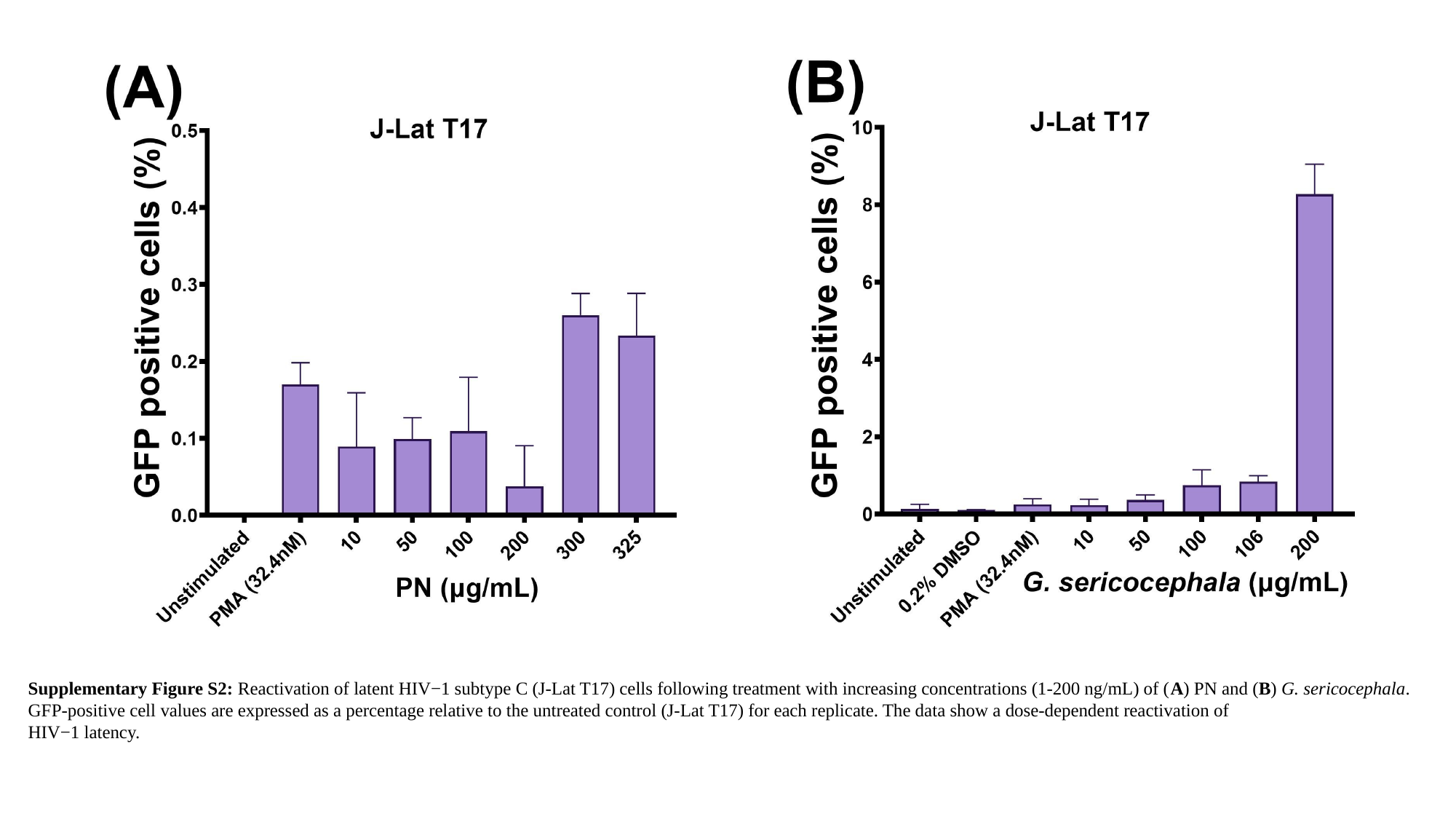

Supplementary Figure S2: Reactivation of latent HIV−1 subtype C (J-Lat T17) cells following treatment with increasing concentrations (1-200 ng/mL) of (A) PN and (B) G. sericocephala.
GFP-positive cell values are expressed as a percentage relative to the untreated control (J-Lat T17) for each replicate. The data show a dose-dependent reactivation of
HIV−1 latency.

## Slide 3
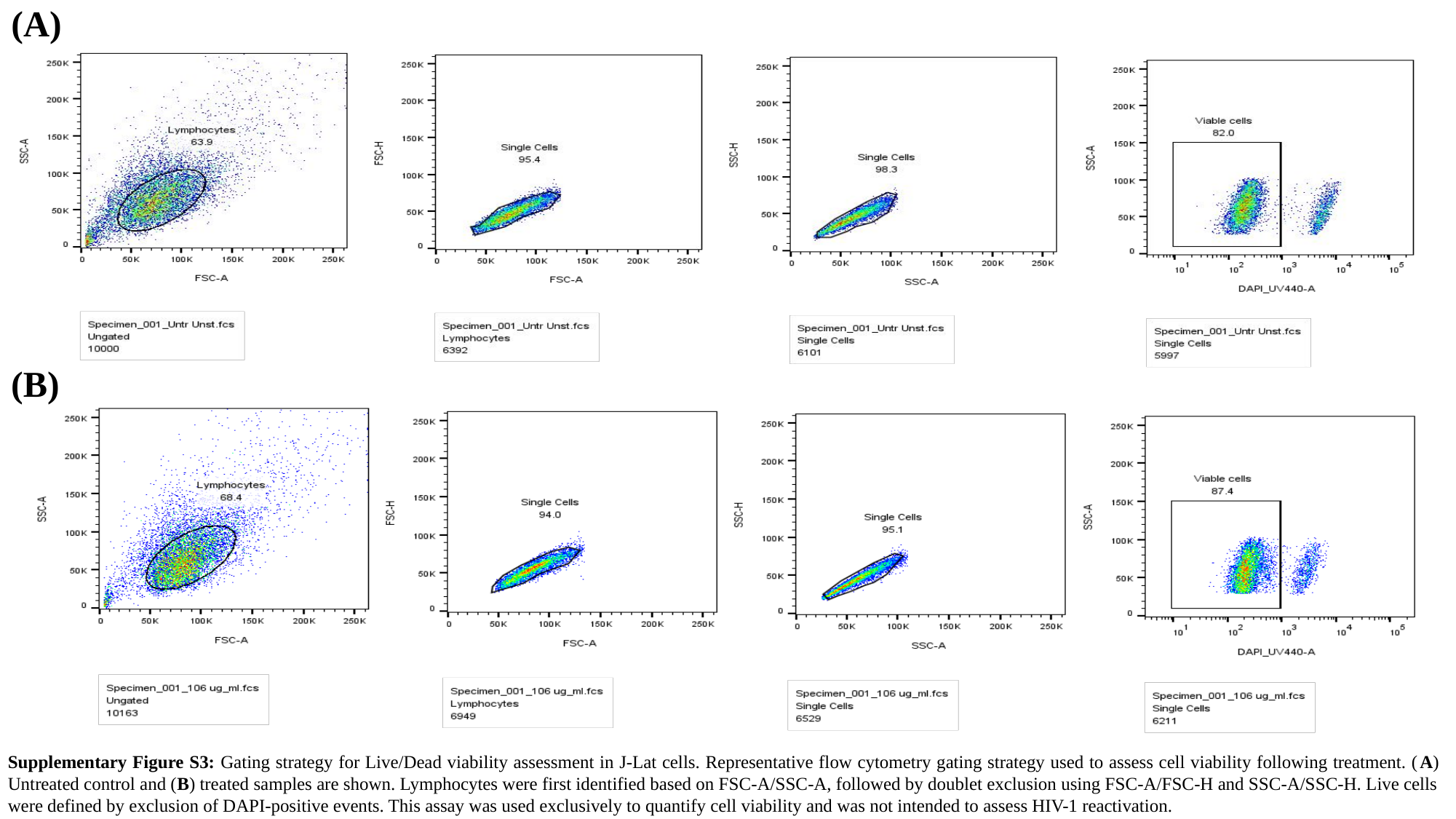

(A)
(B)
Supplementary Figure S3: Gating strategy for Live/Dead viability assessment in J-Lat cells. Representative flow cytometry gating strategy used to assess cell viability following treatment. (A) Untreated control and (B) treated samples are shown. Lymphocytes were first identified based on FSC-A/SSC-A, followed by doublet exclusion using FSC-A/FSC-H and SSC-A/SSC-H. Live cells were defined by exclusion of DAPI-positive events. This assay was used exclusively to quantify cell viability and was not intended to assess HIV-1 reactivation.

## Slide 4
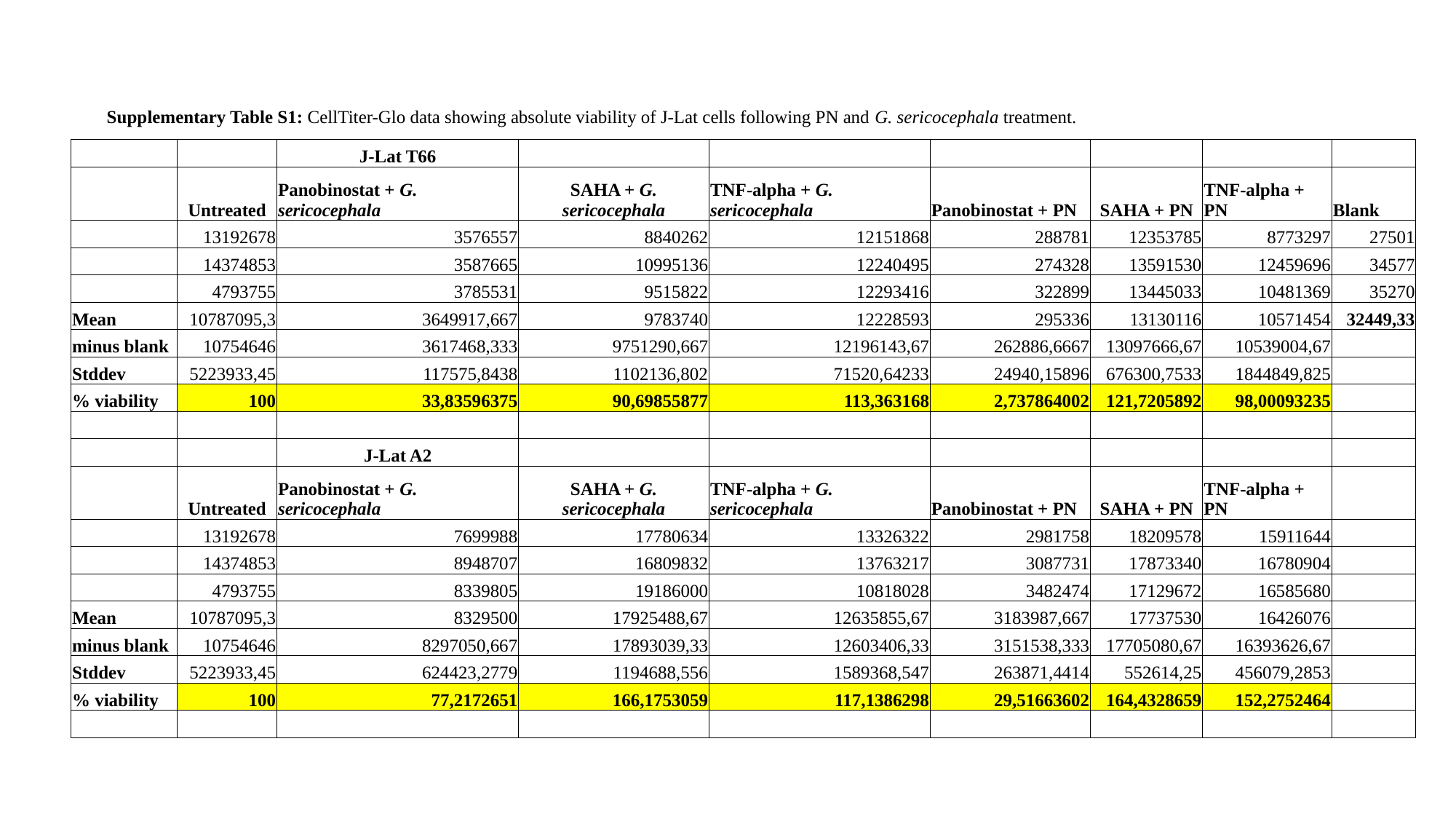

Supplementary Table S1: CellTiter-Glo data showing absolute viability of J-Lat cells following PN and G. sericocephala treatment.
| | | J-Lat T66 | | | | | | |
| --- | --- | --- | --- | --- | --- | --- | --- | --- |
| | Untreated | Panobinostat + G. sericocephala | SAHA + G. sericocephala | TNF-alpha + G. sericocephala | Panobinostat + PN | SAHA + PN | TNF-alpha + PN | Blank |
| | 13192678 | 3576557 | 8840262 | 12151868 | 288781 | 12353785 | 8773297 | 27501 |
| | 14374853 | 3587665 | 10995136 | 12240495 | 274328 | 13591530 | 12459696 | 34577 |
| | 4793755 | 3785531 | 9515822 | 12293416 | 322899 | 13445033 | 10481369 | 35270 |
| Mean | 10787095,3 | 3649917,667 | 9783740 | 12228593 | 295336 | 13130116 | 10571454 | 32449,33 |
| minus blank | 10754646 | 3617468,333 | 9751290,667 | 12196143,67 | 262886,6667 | 13097666,67 | 10539004,67 | |
| Stddev | 5223933,45 | 117575,8438 | 1102136,802 | 71520,64233 | 24940,15896 | 676300,7533 | 1844849,825 | |
| % viability | 100 | 33,83596375 | 90,69855877 | 113,363168 | 2,737864002 | 121,7205892 | 98,00093235 | |
| | | | | | | | | |
| | | J-Lat A2 | | | | | | |
| | Untreated | Panobinostat + G. sericocephala | SAHA + G. sericocephala | TNF-alpha + G. sericocephala | Panobinostat + PN | SAHA + PN | TNF-alpha + PN | |
| | 13192678 | 7699988 | 17780634 | 13326322 | 2981758 | 18209578 | 15911644 | |
| | 14374853 | 8948707 | 16809832 | 13763217 | 3087731 | 17873340 | 16780904 | |
| | 4793755 | 8339805 | 19186000 | 10818028 | 3482474 | 17129672 | 16585680 | |
| Mean | 10787095,3 | 8329500 | 17925488,67 | 12635855,67 | 3183987,667 | 17737530 | 16426076 | |
| minus blank | 10754646 | 8297050,667 | 17893039,33 | 12603406,33 | 3151538,333 | 17705080,67 | 16393626,67 | |
| Stddev | 5223933,45 | 624423,2779 | 1194688,556 | 1589368,547 | 263871,4414 | 552614,25 | 456079,2853 | |
| % viability | 100 | 77,2172651 | 166,1753059 | 117,1386298 | 29,51663602 | 164,4328659 | 152,2752464 | |
| | | | | | | | | |

## Slide 5
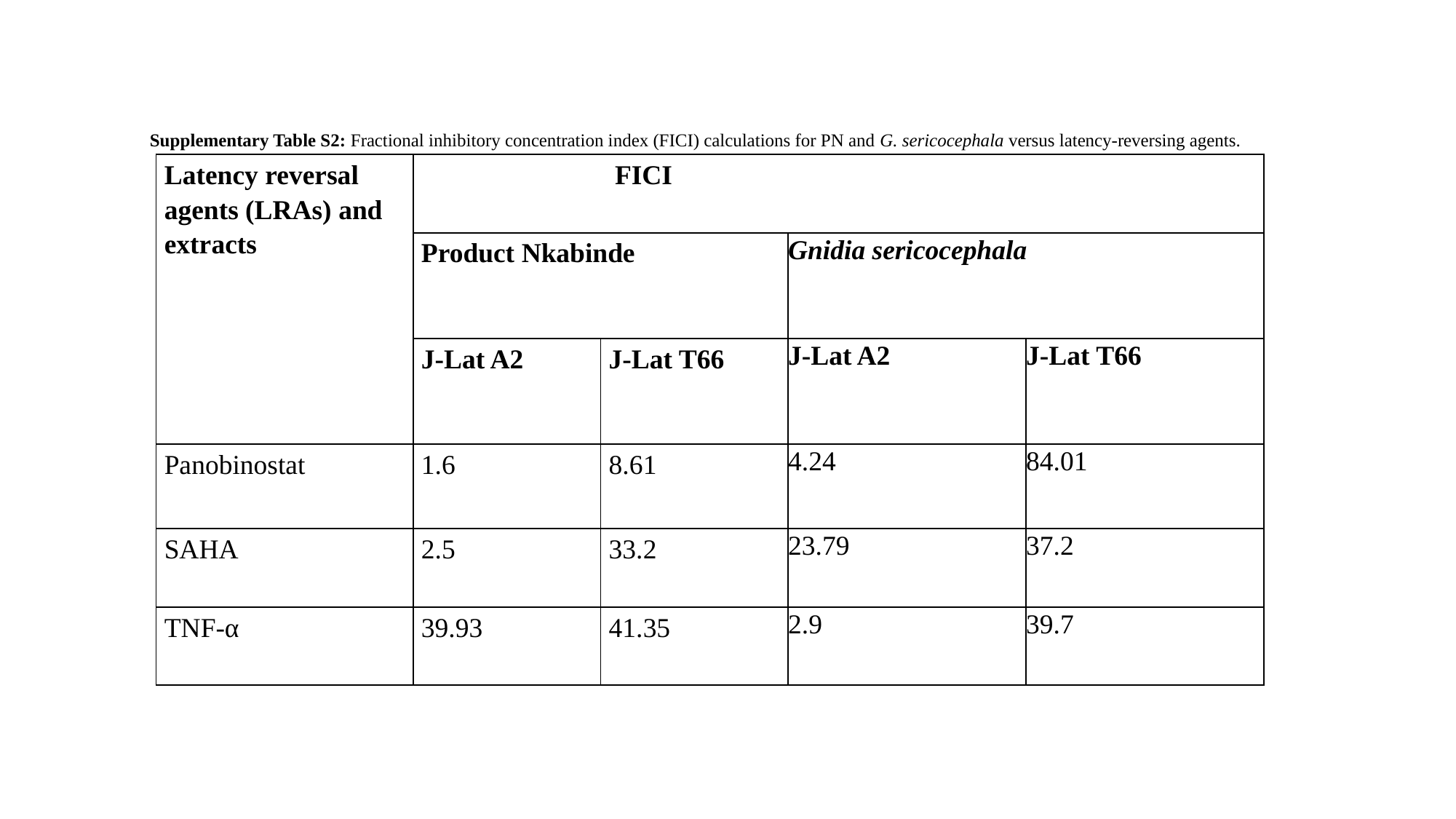

Supplementary Table S2: Fractional inhibitory concentration index (FICI) calculations for PN and G. sericocephala versus latency-reversing agents.
| Latency reversal agents (LRAs) and extracts | FICI | | | |
| --- | --- | --- | --- | --- |
| | Product Nkabinde | | Gnidia sericocephala | |
| | J-Lat A2 | J-Lat T66 | J-Lat A2 | J-Lat T66 |
| Panobinostat | 1.6 | 8.61 | 4.24 | 84.01 |
| SAHA | 2.5 | 33.2 | 23.79 | 37.2 |
| TNF-α | 39.93 | 41.35 | 2.9 | 39.7 |
